# ^1^H nuclear magnetic resonance spectroscopy characterisation of metabolic phenotypes in the medulloblastoma of the SMO transgenic mice

**DOI:** 10.1038/sj.bjc.6605890

**Published:** 2010-09-14

**Authors:** S K Hekmatyar, M Wilson, N Jerome, R M Salek, J L Griffin, A Peet, R A Kauppinen

**Affiliations:** 1 Department of Radiology, Biomedical NMR Research Center, Dartmouth College, 706 Vail, Hanover, NH 03755, USA; 2 School of Cancer Sciences, University of Birmingham, Birmingham, UK; 3 Department of Biochemistry, University of Cambridge, Cambridge, UK

**Keywords:** ^1^H MRS, medulloblastoma, smoothened receptor, transgenic mice, cerebellum, metabolites

## Abstract

**Background:**

Human medulloblastomas exhibit diverse molecular pathology. Aberrant hedgehog signalling is found in 20–30% of human medulloblastomas with largely unknown metabolic consequences.

**Methods:**

Transgenic mice over-expressing smoothened (SMO) receptor in granule cell precursors with high incidence of exophytic medulloblastomas were sequentially followed up by magnetic resonance imaging (MRI) and characterised for metabolite phenotypes by ^1^H MR spectroscopy (MRS) *in vivo* and *ex vivo* using high-resolution magic angle spinning (HR-MAS) ^1^H MRS.

**Results:**

Medulloblastomas in the SMO mice presented as T_2_ hyperintense tumours in MRI. These tumours showed low concentrations of *N*-acetyl aspartate and high concentrations of choline-containing metabolites (CCMs), glycine, and taurine relative to the cerebellar parenchyma in the wild-type (WT) C57BL/6 mice. In contrast, ^1^H MRS metabolite concentrations in normal appearing cerebellum of the SMO mice were not different from those in the WT mice. Macromolecule and lipid ^1^H MRS signals in SMO medulloblastomas were not different from those detected in the cerebellum of WT mice. The HR-MAS analysis of SMO medulloblastomas confirmed the *in vivo* ^1^H MRS metabolite profiles, and additionally revealed that phosphocholine was strongly elevated in medulloblastomas accounting for the high *in vivo* CCM.

**Conclusions:**

These metabolite profiles closely mirror those reported from human medulloblastomas confirming that SMO mice provide a realistic model for investigating metabolic aspects of this disease. Taurine, glycine, and CCM are potential metabolite biomarkers for the SMO medulloblastomas. The MRS data from the medulloblastomas with defined molecular pathology is discussed in the light of metabolite profiles reported from human tumours.

Medulloblastoma is the most common malignant brain tumour of childhood accounting for 25% of all paediatric brain tumours (Taylor *et al*, 2005). It is an aggressive tumour, which commonly invades surrounding structures with metastases being present in over 90% of mortalities (Allen and Epstein, 1982). Medulloblastoma belongs to a group of tumours known as primitive neuroectodermal tumours (PNETs), as they primarily consist of cells that have an undifferentiated neuroepithelial, or stem-cell like morphology (Hart and Earle, 1973). The tumour has the characteristic of being highly cellular, with cells showing little cytoplasm and nuclei with a high affinity for haematoxylin (McLendon *et al*, 1999); so called ‘small round blue cells’. Diagnosis is usually made from this appearance, and further corroborated with immunostaining for neuronal markers, such as synaptophysin.

Although histopathology is regarded as the ‘gold standard’ diagnostic investigation in clinical practise, other techniques which measure the molecular profiles of tumour tissue may offer complementary information on tissue function. Gene expression profiling has been shown to be capable of detecting different subtypes of medulloblastoma, leading to the discovery of prognostic markers (Pomeroy *et al*, 2002). Of particular interest are non-invasive methods, such as *in-vivo* ^1^H magnetic resonance spectroscopy (MRS), which can measure the concentrations of small molecules present in tissues. *In vivo* ^1^H MRS has identified biomarkers which can be used to identify medulloblastoma tumours which are likely to metastasise, (Peet *et al*, 2007a) and these could potentially be used to identify the pathways which may be suitable targets for drugs (Griffin and Shockcor, 2004). A complementary technique to *in vivo* ^1^H MRS is high-resolution magic angle spinning (HR-MAS), which allows a larger number of metabolites to be resolved as well as the accurate detection of lower concentration metabolites from biopsy tissue and cells grown in culture (Peet *et al*, 2007b; Wilson *et al*, 2009a). This technique may be used to validate findings from *in vivo* MRS, determine the contributions of individual metabolites which can only be quantified in combination *in vivo* (Griffin *et al*, 2003; Davies *et al*, 2008) and additionally may reveal low concentration biomarkers which are difficult to measure *in vivo*.

In addition to diagnosis and characterisation, *in vivo* ^1^H MRS is well suited to assessing tumour response to treatment (Hakumäki and Kauppinen, 2000; Nelson and Cha, 2003). It has been shown that in favourable anti-cancer drug treatment and/or radiation therapy of adult glioblastomas ^1^H MRS detectable metabolites strongly decline (Nelson and Cha, 2003). Hakumäki *et al* (1999) showed that ^1^H MRS can detect an increase in lipid signals as tumours undergo gene therapy-induced cell death in a rat glioma model. In addition high, lipids have been associated with high-grade brain tumours in adults (Kuesel *et al*, 1994) and children (Astrakas *et al*, 2004).

Of further importance to the understanding of medulloblastoma, and in particular their response to treatment, is the development of a realistic animal model. Preclinical testing of new agents is a pre-requisite for human use and it is highly desirable for this to be performed in an animal model. New molecularly targeted agents are the focus of most anticancer drug development at present but conventional imaging may provide a poor marker of response (O'Connor *et al*, 2008). The development and validation of novel imaging techniques in tandem with the new agent during the preclinical phase would provide major benefits for the incorporation of this methodology in early phase clinical trials (O'Connor *et al*, 2008). In addition, the study of paediatric brain tumour in appropriate animal models should provide new therapeutic insights into the human disease and might yield important pathophysiological findings. In this study, a novel medulloblastoma mouse model, the smoothened (SMO) mouse, developed by Hatton *et al* (2008), is investigated using MR imaging (MRI) and ^1^H MRS methods, both *in vivo* and *ex vivo*, to characterise its morphologic and molecular features. The SMO mouse model has high levels of the activated form of SMO receptor in Sonic hedgehog (Shh) signalling pathway, which results in a high rate of medulloblastoma formation and early cerebellar hyperproliferation (Hatton *et al*, 2008). The Shh pathway is crucial for normal development of the cerebellum. The mutations in the patched (*Ptch*) gene in the Shh pathway contribute to SMO mice developing subclinical medulloblastoma by two months of age. The close adherence to human pathology, high incidence, a greater latency period, and early onset of tumours thus make SMO mice an efficient model for preclinical studies and for further investigating the series of events leading to tumour formation and progression, however, the metabolic profile of these tumours has not been established.

In this work, we have used MRI and ^1^H MRS to determine the metabolite profile in the SMO medulloblastomas *in vivo* and *ex vivo* allowing a comparison with childhood medulloblastomas and establishing a test bed for ^1^H MRS biomarkers from this malignant brain tumour with defined molecular pathology. ^1^H MRS was used to identify the presence of the tumours, to follow their progression in contrast to MRI method, because of the weak contrast enhancement in the tumour.

## Materials and methods

The ND2:SmoA1 mouse line (the SMO mouse) was a generous gift from Fred Hutchinson Cancer Research Center (Seattle, WA, USA) and was generated as previously described (Hallahan *et al*, 2004; Hatton *et al*, 2008). Adult homozygous SMO mice were maintained under sterile conditions in accordance with the Institutional Animal Care and Use Committee at the Dartmouth Medical School. Wild-type (WT) male C57BL6 mice were purchased from Charles River Laboratories. All mice were monitored on a regular basis for signs of illness or tumour effects, such as symptoms of protruded head and lethargy.

*In vivo* MRI and MRS were performed on the SMO and WT mice. SMO mice (*n*=19) ranging from 55 to 229 days and WT of over 120 days of age were used for the study (weight ranging from 20 to 30 g). All MR experiments were performed on a 7T/21 cm magnet (Magnex Scientific, Abingdon, UK) equipped with an imaging gradient set (Resonance Research Inc, Billerica, MA, USA), interfaced to a Varian INOVA console (Varian, Palo Alto, CA, USA). A single tuned one turn surface coil with diameter of 15 mm diameter was used in transmit/receive mode. The mice were anaesthetised with isoflurane (1–1.5 volume % in 70 : 30 O_2_:air) with a nosecone and animal torso was placed on a thermostated water circulating heating element at 37°C for the duration of MR scans. In all MR experiments, a multislice multiecho sequence was used to acquire T_2_-weighted images and to position the voxel for ^1^H MRS studies with following parameters: time to repetition (TR) 3 s, echo train length 8, echo spacing 15 ms, effective echo time (TE) 60 ms, number of phase encoding steps 128, FOV 32 × 28 mm^2^, sagittal orientation, slice thickness 0.75 mm, number of transients 4. The multi slice spin echo T_1_-weighted images before and 15 min after contrast agent injection (Magnevist, Bayer Inc., Wayne, NY, USA; 0.2 mmol kg^–1^ i.p.) were acquired with same field of view and thickness but with TR of 0.7 s, TE of 9 ms.

For single voxel ^1^H localised MRS shimming was performed using FASTESTMAP (Gruetter, 1993; Gruetter and Tkac, 2000), resulting in a 10–17 Hz full width at half height of the water resonance in an 8 *μ*l voxel (2 × 2 × 2 mm^3^). Voxels were centred to the T_2_ hyperintense tumour in the SMO mice or in the cerebellum of WT mice (and those SMO mice showing normal T_2_ MRI) to minimise partial volume effects. Localisation by adiabatic selective refocusing (Garwood and DelaBarre, 2001) technique was used for single voxel with TR of 3 s and TE of 26 ms, with 256 signal averages. The water signal was efficiently suppressed by variable power RF pulses with optimised relaxation delays (Tkac *et al*, 1999). Unsuppressed water signal was acquired at the end of the experiments for quantification of metabolites (eight averages). The receiver gain and signal averages were taken into account for quantification.

### Magic angle spinning

Tumour and brain tissues were stored at −80°C until analysis, thawed on dry ice, and cut to approximately 20 mg specimens for ^1^H HR-MAS. The tissue was then placed into a 30 *μ*l Kel-F rotor insert and weighed. 3 *μ*l 3-(trimethylsilyl) propionic-2,2,3,3-d_4_ acid (sodium salt) was dissolved in D_2_O at a concentration of 5 mM and added to the rotor insert. The remaining volume of the rotor was filled with D_2_O to ensure consistent spinning for each sample.

The ^1^H HR-MAS was performed on a Bruker 500 MHz vertical-bore spectrometer using a 4 mm HRMAS ^1^H-^13^C NMR probe with a z-gradient (Bruker UK Limited, Coventry, UK) with an Advance III console running TOPSPIN 2.1 software. The sample was spun at 5000 Hz at a temperature of 27°C determined by methanol calibration. A 1D NOESY pre-saturation pulse sequence was used, which consisted of a single 90° pulse preceded by NOESY water pre-saturation. The 90° pulse was followed by the acquisition of 32 K complex points acquired with a spectral width of 16 p.p.m. The sequence had a repetition time of 4 s and 64 or 128 scans were acquired depending on the sample size.

### Histology

A subgroup of animals were killed at the end of scan and the brain tissue was removed from skull and rinsed in cold phosphate-buffered saline (PBS) and fixed in 4% paraformaldehyde in PBS solution and kept for overnight. The fixated brains were cryoprotected with 20% glycerol solution in 20 mM PBS for 24 h. The brains were frozen in a dry ice and stored at −70°C until sectioned.

Serial sagittal 10–15-*μ*m cryostat sections, corresponding to MR images, were mounted on glass slides and stained with hematoxylin and eosin (Fisher, Pittsburgh, PA, USA) using standard methods. Stained sections were examined with a Nikon E800 microscope (Nikon, Melville, NY, USA). Histological pictures were viewed using a colour camera and software (version 3.0, Diagnostic Instruments, Sterling Heights, MI, USA).

### Data analysis

#### Magnetic resonance imaging

The Aedes routine (http://aedes.uku.fi) under Matlab platform (Mathworks Inc, Bolder, CO, USA) was used to determine length of cerebrum and cerebellum at midline as well as size of 4th ventricle from multislice sagittal T_2_ scans.

#### Spectroscopic analysis

*In vivo* and HR-MAS ^1^H MR spectra were automatically phased and referenced to the creatine peak at 3.03 p.p.m. before being analysed using the TARQUIN algorithm for *in vivo* MRS as described by workers Wilson *et al* (2010). Simulated basis sets were generated assuming ideal pulses, and metabolite parameters were taken from (Govindaraju *et al*, 2000). For HR-MAS data metabolite chemical shifts were manually optimised using wxNUTS (Acorn NMR Inc, CA, USA) to account for the difference in temperature between *in vivo* and *in vitro*. *In vivo* metabolite concentrations were determined from the unsuppressed water experiment, assuming an MR visible water molarity of 35 880 mM. Metabolite quantities are expressed as ratios to creatine for the HR-MAS data as this metabolite was found to have a stable concentration across samples.

#### Statistical analysis

Metabolite quantities were imported into the R statistics package (Team, 2009) and the Student's *t*-test and one-way analysis of variance methods were used to determine differences between the WT and SMO mice.

## Results

**Figure 1:**
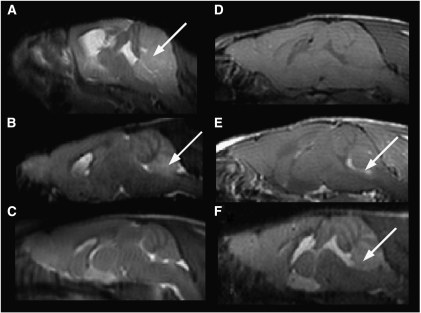
T_2_ MR images from the SMO (**A** and **B**) and WT (**C**) mice. Arrows point to T_2_ hyperintensity in the cerebellum because of medulloblastoma tumours. T_1_-weighted MR images from mouse in panel B before (**D**) and 15 min after (**E**) i.p. injection of Magnevist. An arrow in (**E**) shows the signal enhancement in the tumour periphery. A T_2_-weighted image from the mouse in (**D** and **E**) is shown in (**F**). Details for MRI given under Materials and Methods section.

The sagittal T_2_-weighted images from two SMO mice show mass that covers a large volume of the cerebellum (Figure 1A) or presents as a solitary tumour in the base of the cerebellum (Figure 1B). The foliage pattern of cerebellum was absent in the T_2_ hyperintense tumours. In the mouse in Figure 1A, a severe hydrocephalus with gross brain deformity is evident. A total of 19 SMO mice were scanned with three types of cerebellar appearances in T_2_-weigthed RARE MRI as follows: (a) no obvious abnormality (*n*=10), the age of these mice varied from 55 to 220 days; (b) focal hyperintense tumour (*n*=2, Figure 1B), these mice were 119 and 180 days old; and (c) widespread hyperintensity and large-sized ceberellum (*n*=7, Figure 1A), the age range of these mice was from 69 to 229 days. A T_1_-weighted image obtained before (Figure 1D) and after contrast agent injection (Figure 1E) show signal enhancement in the periphery of the tumour (for a T_2_-weighted MRI of this mouse, see Figure 1F) that was commonly observed in the SMO medulloblastomas studied.

**Figure 2:**
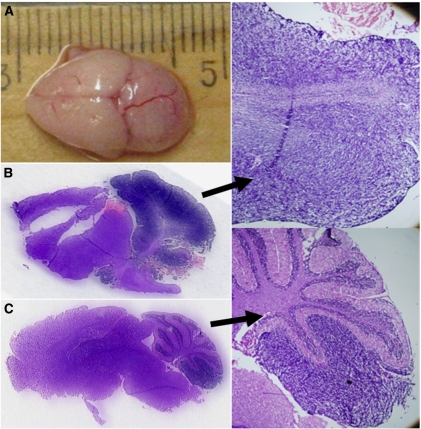
Macroscopic CNS phenotype of a SMO mouse (**A**), and hematoxylin and eosin sections from a widespread (**B**) and focal (**C**) medulloblastomas with a × 3 enlargement on right of them.

**Figure 3:**
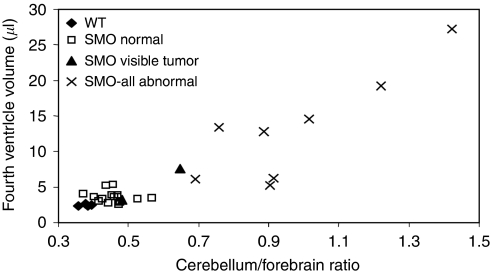
A plot between the cerebellum-to-forebrain length ratio and the volume of the fourth ventricle from WT and SMO mice with and without T_2_ MRI abnormality.

A typical macroscopic brain phenotype of SMO mice is displayed (Figure 2A), where the cerebellum is grossly enlarged approaching the size of the forebrain. This mouse showed widespread T_2_ hyperintensity in the cerebellum with hydrocephalus. A plot between cerebellum-to-forebrain length ratio and the volume of fourth ventricle shows that in a large number of SMO mice both the ratio and the fourth ventricle were higher than in WT mice (Figure 3). Histological sections for focal (Figure 2B) and uniform (Figure 2C and D) medulloblastomas are displayed. The histological slices showed high cell density in the external granular layer in the cerebellum. Small dark and dense cells that are stained strongly with hematoxylin, with disappearance of foliage pattern in MRI images are the microscopic hallmarks indicative of medulloblastomas.

**Figure 4:**
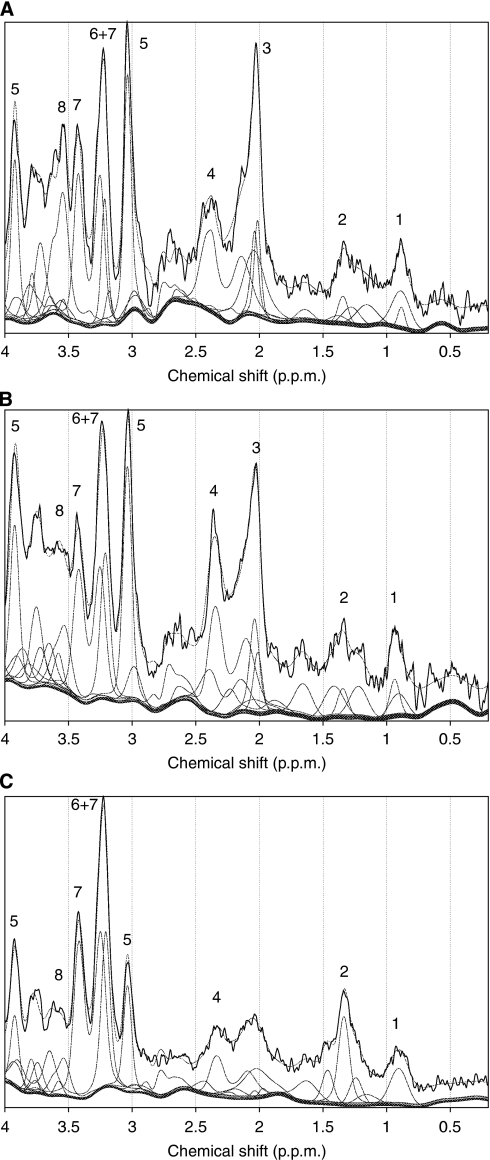
^1^H MRS *in vivo* spectra from and (**A**) WT mouse cerebellum, (**B**) SMO mouse cerebellum without T_2_ hyperintense lesion and (**C**) SMO mouse cerebellum with a T_2_ hyperintense tumour. Peak assignments are as follows: (1) lipids and macromolecules at 0.9 p.p.m., (2) lipids and macromolecules at 1.3 p.p.m.+lactate, (3) *N*-acetyl aspartic acid+*N*-acetyl aspartylglutamic acid, (4) glutamate+glutamine, (5) creatine, (6) choline containing metabolites, (7) taurine, (8) glycine+myo-inositol.

Typical ^1^H MR spectra from WT cerebellum (a), SMO mouse cerebellum with (b) and without (c) T_2_ MRI hyperintense tumour are shown (Figure 4). It is evident that the ^1^H MR spectrum of a tumour in a SMO mouse has a very small resonance associated with N-acetyle aspartate (NAA) and grossly elevated choline-containing metabolite (CCM) and taurine peaks, but normal appearing creatine resonance. Interestingly, the chemical shift region at 1.3 and 0.9 p.p.m. in the spectrum from the SMO medulloblastoma shows only weak signal from lipids, lactate and macromolecules. Quantitative metabolite data are shown in Table 1. It is evident that NAA in medulloblastomas (pooled MRS data from all eight SMO mice with pathological cerebellum in T_2_ MRI) is severely reduced, whereas taurine, glycine, and CCM are highly elevated. Lactate, macromolecules and lipids in the chemical shift region between 0.9 and 1.4 p.p.m. are similar in medulloblastomas and WT mice. In the cerebella of the SMO mice without MRI abnormality, all metabolites, macromolecules and lipids were within the range determined in the cerebella of the WT mice (Table 1).

**Figure 5:**
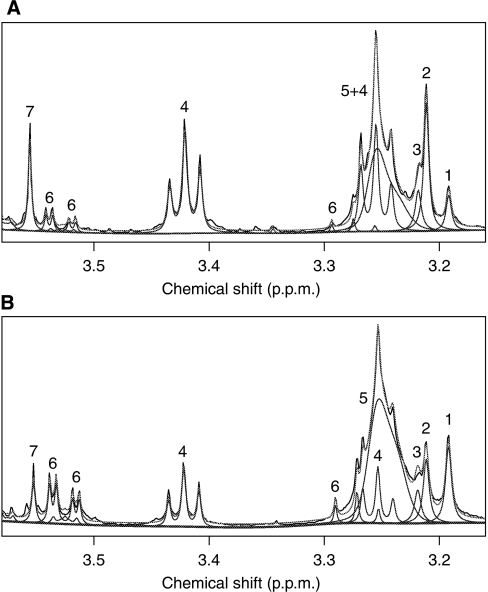
Example ^1^H HR-MAS spectra from (**A**) SMO mouse cerebellum with a T_2_ MRI visible focal lesion and (**B**) a WT mouse cerebellum. Peak assignments are as follows: (1) choline, (2) phosphocholine, (3) glycerophosphocholine, (4) taurine, (5) phosphatidylcholine, (6) myo-inositol and (7) glycine.

Example ^1^H HR-MAS spectra from SMO mouse with T_2_ MRI-detected tumour (a) and WT mouse (b) are shown (Figure 5). The increased resolution of HR-MAS confirms the assignments and trends detected *in vivo* and provides additional metabolic information. The three choline-containing compounds are clearly separable and it is clear that the increase in CCM seen in the SMO medulloblastomas *in vivo* are as a result of an increase in phosphocholine (PC) with glycerophoshocholine remaining stable, a significant reduction in choline is also shown. As shown in Table 1, total creatine concentration *in vivo* was not different between the animals and therefore, we used creatine as a reference for HR-MAS. In addition to changes in CCM, increases in taurine, glycine, and NAA accompanied with decreases in myo-inositol and *γ*-amino butyrate (GABA) are apparent from the spectra. Quantitative data for metabolites are shown in Table 2.

## Discussion

Mouse models that mimic molecular pathology of human cancers provide a tremendous benefit towards molecular understanding of tumour biology. There are more than 15 genetically engineered mouse models for medulloblastoma available for preclinical studies (Goodrich *et al*, 1997; Fomchenko and Holland, 2006; Hatton *et al*, 2006). Very few of these mouse models generate spontaneous tumours, however, and tumours may differ histologically and genetically from human medulloblastomas. To generate a closely human medulloblastoma resembling model Hatton *et al* (2008) have cloned the SMO receptor and developed the ND2:SmoA1 transgenic mouse with spontaneous medulloblastomas sharing several characteristics of human medulloblastoma. These include histopathology of exophytic medulloblastoma, leptomeningeal spread and clinical presentation characterised by ataxia and hydrocephalus. The adherence to human pathology, high incidence, a shorter latency period, and early onset of tumours thus make the SMO mice an excellent animal model for preclinical studies of medulloblastomas.

Our data show that the SMO medulloblastomas resemble closely human medulloblastomas in terms of T_2_ hyperintense appearance. The ^1^H MRS evaluation of genetically engineered medulloblastoma model has not been reported before. The evaluation of brain tumours by means of ^1^H MRS has mainly relied upon metabolite ratios such as NAA/CCM, NAA/creatine, or CCM/creatine. There are several MRS studies involving the human medulloblastoma in children reported elsewhere (Girard *et al*, 1998; Tzika *et al*, 2002; Astrakas *et al*, 2004; Panigrahy *et al*, 2006; Davies *et al*, 2008). Human studies have shown that the primary tumours of metastatic medulloblastoma have increased levels of CCM and low level of lipids (Wilson *et al*, 2009b). Wilson *et al* (2009b) have reported that there was a good correlation between metabolite quantities detected by *ex vivo* ^1^H HR-MAS and *in vivo* ^1^H MRS data. We found that the medulloblastomas in the SMO mice have decreased NAA and elevated CCMs, taurine, and glycine. This resembles closely the metabolic pattern observed in human low grade medulloblastomas with heterogeneous molecular pathology (Peet *et al*, 2004). Low levels of NAA and GABA (by HR-MAS *ex vivo*) in the SMO medulloblastomas probably reflect loss of the neuronal phenotype by the transformed granule cells.

Given that the majority of the SMO mice with T_2_ MRI hyperintense tumours were in their adulthood, it is pertinent to compare metabolite profiles detected in mice with those reported from human adult medulloblastomas and/or PNETs. *In vitro* ^1^H MRS analysis has revealed high concentrations of total choline-containing phospholipids in extracts from adult medulloblastomas (Srivastava *et al*, 2010). Using *in vivo* ^1^H MRS, Majos *et al* (2002) found that adult medulloblastomas have low NAA and creatine, but elevated CCM and lactate/lipids. More recently a short TE ^1^H MRS study (Moreno-Torres *et al*, 2004) showed high taurine concentration in adult medulloblastomas. In the light of the present knowledge, the SMO and adult medulloblastomas share metabolite similarities such as high CCM and taurine, and low NAA, but levels of creatine and lactate/lipids in these tumours may be at variance. Clearly, more data are required from adult medulloblastomas to allow for full comparison of ^1^H MRS metabolite profiles with childhood tumours or with any preclinical medulloblastoma model.

A highly elevated taurine was observed in posterior fossa tumour in children (Schneider *et al*, 2007). In agreement with previous reports (Schneider *et al*, 2007; Davies *et al*, 2008), we have found a consistent and elevated resonance from the taurine peak at 3.3 p.p.m. which is probably indicating the aggressive behaviour of medulloblastoma. Taurine is an aminosulfonic acid, and is abundant in the developing cerebellum and outermost layer of the cerebellum. It has been postulated that increased taurine is associated with an increased cellular proliferation and aggressive nature of tumour (Wilson *et al*, 2009b). These findings underline the importance of taurine and other metabolites together, as imaging biomarkers of higher malignancy in medulloblastomas. More recently, taurine was associated with apoptotic activity in malignant adult brain tumours (Opstad *et al*, 2009).

In this study, it was shown *ex vivo* by HR-MAS that PC is the main contributor to the elevated CCM peak seen *in vivo*. An elevation of PC in medulloblastoma has also been confirmed in several other studies using HR-MAS (Tugnoli *et al*, 2005; Wilson *et al*, 2009b). A similar increase in PC has been shown to be related to the expression of the *MYCN* oncogene in neuroblastoma, which are histologically very similar to medulloblastoma (Peet *et al*, 2007b). The importance of choline metabolism has been related to tumour growth in astrocytic tumours (Usenius *et al*, 1994) and in numerous other studies reviewed by Podo (1999). This study is also in agreement with the observation that glycine is elevated in medulloblastoma (Wilson *et al*, 2009b) and supports recent evidence that it may be a potential ^1^H MRS biomarker of malignancy in childhood (Davies *et al*, 2010) and adult brain tumours (Righi *et al*, 2010).

Lipid levels, as detected by ^1^H MRS, are not significantly high in the SMO medulloblastomas, however, large variation in lipid levels of orthotopic medulloblastomas *in vivo* has been reported (Rosol *et al*, 2009). It is commonly accepted that ^1^H MRS detected lipids are localised in cytoplasmic lipid vesicles (Callies *et al*, 1993; Hakumäki *et al*, 1998). Elevated ^1^H MRS lipid signals are associated with high degree of malignancy in adult (Negendank and Sauter, 1996; Murphy *et al*, 2003) and paediatric brain tumours (Astrakas *et al*, 2004; Albers *et al*, 2005). MRS lipids are associated with cell death in tumours either through apoptosis (Hakumäki and Kauppinen, 2000) or necrosis (Kuesel *et al*, 1994). Recently, MRS lipids were linked to tissue hypoxia in tumours (Zoula *et al*, 2003). Low levels of ^1^H MRS detected lipids in the SMO medulloblastomas, which are highly malignant with poor prognosis, are an unexpected observation in the light of data from other high-grade brain tumours. These data indicate that these medulloblastomas may be well perfused, without significant hypoxia and cell death.

In conclusion, the genetically engineered SMO medulloblastoma shares several MRI properties with human medulloblastomas. Interestingly, we found that the metabolites pattern of the SMO medulloblastoma resembles that of human medulloblastomas which have already metastasised. This animal model is a suitable tool for further experimental research including treatment studies. This study suggests that ^1^H MRS will be of potential clinical value primarily in differential diagnostic considerations.

## Figures and Tables

**Table 1:** Metabolite concentrations (mM) determined from ^1^H MR spectra with the internal water as a reference

		**SMO mice**	**SMO mice**		
**Metabolite**	**Wild type (*n*=5)**	**Normal T_2_ MRI (*n*=8)**	**T_2_ lesion (*n*=10)**	***t*-Test**	**ANOVA**
Alanine	1.8±1.7	0.96±1.11	0.91±1.68	0.38	0.51
Aspartate	8.6±5.8	6.8±6.6	12.9±7.8	0.28	0.19
Creatine	10.6±2.5	8.3±2.1	8.5±2.3	0.16	0.16
*γ*-Aminobutyric acid	2.5±4.0	6.6±4.8	7.3±7.0	0.14	0.30
Glycerophosphocholine	1.8±1.1	2.1±0.6	2.9±1.4	0.15	0.13
Glucose	5.9±3.6	3.9±2.1	3.8±2.8	0.31	0.35
Glutamine	11.4±10.9	9.3±6.4	9.7±7.4	0.77	0.89
Glutamate	10.6±9.3	10.1±5.8	6.1±3.7	0.35	0.31
Glycine	0.73±1.13	2.4±3.3	5.0±2.7	0.003	0.04
Guanidinoacetate	0.85±1.37	0.71±1.09	1.7±1.8	0.34	0.32
Myo-inositol	9.4±2.2	6.4±3.3	6.0±4.7	0.11	0.26
Lactate	3.4±1.8	1.3±1.3	3.6±4.0	0.90	0.16
*N*-acetylaspartate	2.0±2.5	2.3±1.9	0.35±0.77	0.22	0.07
*N*-acetylaspartylglutamate	4.4±2.9	2.7±2.1	1.0±0.6	0.06	0.02
Phosphocholine	0.38±0.66	0.33±0.51	1.2±1.4	0.16	0.12
Scyllo-inositol	0±0	0.09±0.22	0.46±0.67	0.09	0.11
Taurine	11.6±1.4	10.4±2.1	20.1±4.3	0.001	0.00
Lipid at 1.3 p.p.m.	2.1±1.8	4.3±3.3	3.8±2.2	0.15	0.34
Macromolecule at 0.9 p.p.m.	4.4±2.8	5.3±2.5	4.4±2.4	0.99	0.74
Total choline	2.2±0.6	2.4±0.5	4.2±1.0	0.0011	0.00
Total NAA	6.4±1.1	5.0±1.6	1.30±1.0	0.0000	0.00

Abbreviations: ANOVA=analysis of variance; MR=magnetic resonance; MRI=magnetic resonance imaging; SMO=smoothened.

Values are means±standard deviations. The Student's *t*-test was performed between the wild-type and SMO mice with a T_2_ lesion, ANOVA was performed between the three groups.

**Table 2:** Metabolite quantities as a ratio to creatine determined from ^1^H HR-MAS spectra

		**SMO mice**	**SMO mice**		
**Metabolite**	**Wild type (*n*=4)**	**Normal T_2_ MRI (*n*=7)**	**T_2_ lesion (*n*=4)**	***t*-Test**	**ANOVA**
Alanine	0.10±0.062	0.10±0.03	0.22±0.11	0.10	0.02
Aspartate	0.44±0.12	0.43±0.06	0.30±0.04	0.10	0.04
Choline	0.16±0.03	0.13±0.02	0.11±0.02	0.04	0.03
*γ*-Aminobutyric acid	1.08±0.15	0.88±0.16	0.49±0.21	0.005	0.001
Glycerophosphocholine	0.08±0.02	0.08±0.04	0.09±0.03	0.39	0.73
Glutamine	0.23±0.07	0.23±0.13	0.24±0.15	0.92	0.99
Glutamate	0.70±0.11	0.64±0.08	0.73±0.13	0.80	0.35
Glycine	0.31±0.08	0.24±0.03	0.52±0.15	0.05	0.001
Myo-inositol	0.75±0.14	0.70±0.07	0.41±0.12	0.01	0.001
Lactate	1.15±0.07	1.10±0.14	1.23±0.18	0.42	0.33
*N*-acetylaspartate	0.38±0.00	0.34±0.03	0.18±0.08	0.02	0.0001
*N*-acetylaspartylglutamate	0.001±0.001	0.03±0.04	0±0	0.18	0.28
Phosphocholine	0.15±0.02	0.16±0.02	0.28±0.06	0.03	0.001
Scyllo-inositol	0.004±0.006	0.002±0.002	0.008±0.005	0.33	0.09
Succinate	0.02±0.01	0.02±0.01	0.02±0.001	0.33	0.56
Taurine	0.88±0.11	0.86±0.10	1.79±0.59	0.06	0.001

Abbreviations: ANOVA=analysis of variance; HR-MAS=high-resolution magic angle spinning; MRI=magnetic resonance imaging; SMO=smoothened.

Values are means±standard deviations. The Student's *t*-test was performed between the wild-type and SMO mice with a T_2_ lesion, ANOVA was performed between the three groups.
